# Sure you are ready? Gendered arguments in recruitment for high-status positions in male-dominated fields

**DOI:** 10.3389/fpsyg.2022.958647

**Published:** 2023-01-30

**Authors:** Regina Dutz, Sylvia Hubner-Benz, Franziska Emmerling, Claudia Peus

**Affiliations:** ^1^TUM School of Management, Technical University of Munich, Munich, Germany; ^2^Faculty of Economics and Management, Free University of Bozen-Bolzano, Bolzano, Italy

**Keywords:** STEM professorships, recruitment, gender, heuristics, stereotyping, signaling

## Abstract

Recruitment contexts such as STEM professorships promote clearly defined selection criteria and objective assessment. We illuminate in these contexts, the subjective interpretation of seemingly objective criteria and gendered arguments in discussions of applicants. Additionally, we explore gender bias despite comparable applicant profiles investigating how specific success factors lead to selection recommendations for male and female applicants. Implementing a mixed methods approach, we aim to highlight the influence of heuristics, stereotyping, and signaling in applicant assessments. We interviewed 45 STEM professors. They answered qualitative open-ended interview questions, and evaluated hypothetical applicant profiles, qualitatively and quantitatively. The applicant profiles enabled a conjoint experiment with different applicant attributes varied across the profiles (i.e., publications, willingness to cooperate, network recommendation, and applicant gender), the interviewees indicating scores of selection recommendation while thinking aloud. Our findings reveal gendered arguments, i.e., questioning women potentially fueled by a perception of women’s exceptional status and perceived self-questioning of women. Furthermore, they point to gender-independent and gender-dependent success patterns, thereby to potential success factors particularly for female applicants. We contextualize and interpret our quantitative findings in light of professors’ qualitative statements.

## Introduction

Men continue to occupy most high-status and influential positions in the world of work (see, e.g., Catalyst, 2020a,b; Levanon and Grusky, 2016). A driver of sustained gender inequality are gender biases in recruitment evaluations based on stereotyped beliefs (Heilman, 2012; Koch et al., 2015; Begeny et al., 2020). Clearly defined selection criteria that are objectively assessable have been suggested to counter such gender biases (Heilman, 2012). For example, in academic recruitment for professorships, the strict regulations of public authority and clear output/performance indicators can be seen as largely objective leaving no room for gender-biased interpretation. Yet, particularly in the STEM fields (science, technology, engineering, mathematics) men continue to occupy the majority of professorships (e.g., GWK, 2020; McCullough, 2020). We argue that, even in contexts promoting clearly defined criteria and objective assessment, such as academia, these criteria can be subjectively interpreted and construed differently for men vs. women, leading to gender-biased evaluations (van den Brink and Benschop, 2012; Herschberg et al., 2015). To illuminate gender (in)equality arising from subjective interpretation of seemingly objective criteria, we investigate how gendered arguments find their way into the assessment of applicants for STEM professorships.

In recruitment for STEM professorships, as in recruitment for other high-status jobs in male-dominated fields, stereotypical perceptions of who fits a position favor men (Heilman, 2012; Carli et al., 2016; Dutz et al., 2022). Due to gender stereotypes, women are considered a “risky” option (Fleming Cabrera, 2010). Research on professorial recruitment shows that female applicants are evaluated based on a “proven masculine success model” (van den Brink and Benschop, 2014, p. 17), thus, on different standards than men. Additionally, research shows that academic “excellence,” as halo selection criterion, is a gendered construct and subjectively discussed (van den Brink and Benschop, 2012). Research on heuristics and stereotyping enlightens how various biases influence recruitment; based on stereotyped heuristics, women and men are generally ascribed different qualities, and their behavior is interpreted differently (e.g., Rudman and Phelan, 2008; Heilman, 2012). Stereotype biases further reduce perceived fit, particularly when the stereotypical image of traditional job holders (male professors) does not match the applicant’s gender (Tversky and Kahneman, 1974; Heilman, 1983, 2012). This phenomenon extends to self-assessments as stereotypes influence which qualities women and men under- or overestimate in themselves (Heilman, 2001; Hentschel et al., 2019). Therefore, evaluators may assume that women feel uncomfortable when showing male-typed, i.e., stereotype-inconsistent, behavior to selection committees or in their daily work (e.g., determination or competitiveness; Heilman, 2001; Rudman et al., 2012). We investigate how heuristics and stereotyping contribute to the persistence of gendered arguments.

In the context of STEM professorships, we intend to illuminate how both subjective heuristics (Tversky and Kahneman, 1974; Heilman, 2012) and objectively observable signals (e.g., provided information on applicants’ education or skills; Spence, 1973; Rynes, 1991; Connelly et al., 2011) influence evaluators’ perceptions and thereby discussions in selection committees. It is challenging to precisely disentangle the influence of subjective heuristics and observable signals in recruitment. Clearly defined selection criteria (e.g., publication track record) which applicants can provide objective information on (e.g., in their CV or during interviews) can reduce subjectivity and stereotyping (Nieva and Gutek, 1980; Heilman, 2012). However, although criteria may be clearly defined and information cues on those criteria may be objectively observable, there can be subjective interpretation of selection criteria, applicant signals, or both (see, e.g., van den Brink and Benschop, 2012). For instance, applicants can provide information on their number of publications and emphasize their cooperativeness, while evaluators subjectively assess whether the exact publications reflect a successful publication track record and how cooperation would look like. We investigate the duality of subjective interpretation of seemingly objective criteria.

Investigating the more subjective and the more objective parts of applicant assessments, we also look at how specific signals that are objectively observable are evaluated for women vs. men. Evaluators may vary in their perception of how important a criterion (or signal) is, and this perception may be stereotyped reflecting the gendered success model. That is, we investigate whether success patterns are gendered, i.e., whether some signals may be success factors for women but not for men.

In our mixed-method research we collected qualitative as well as quantitative data from 45 tenured STEM professors in Germany. We conducted interviews and integrated a conjoint experiment. On the one hand, participating professors answered open-ended questions. On the other hand, they qualitatively and quantitatively evaluated hypothetical applicant profiles *via* completing a web-based conjoint experiment while thinking aloud. We inductively coded the interviewees’ verbatim statements in response to the questions and profiles (Gioia et al., 2013; Eisenhardt et al., 2016) and identified emerging themes of gendered arguments. Moreover, we analyzed their quantitative evaluations of the profiles *via* fsQCA (fuzzy set qualitative comparative analysis; Ragin, 2008a) to identify success patterns that led to a high vs. low selection recommendation for male and female applicants.

Our research makes three main contributions to the literature. First, building on prior findings of gendered discussions of applicants for professorships (e.g., van den Brink and Benschop, 2012, 2014), we update and extend the knowledge of the persistence and mechanisms of gendered arguments. We show how gendered arguments build barriers for women’s advancement, specifically into STEM professorships, which are high-status positions in male-dominated fields and therefore have a strong male stereotype (Carli et al., 2016; Dutz et al., 2022). We highlight that gendered arguments influence evaluations despite desired objectivity in applicant assessments. We delineate different forms of gendered arguments related to other-stereotyping, perceptions of applicants’ self-stereotyping (Heilman, 2001; Hentschel et al., 2019), traditional social roles (Eagly, 1987; Eagly and Wood, 2012), and inclusion concerns based on “chilly climate” perceptions (e.g., Hinsley et al., 2017).

Second, we provide a more nuanced understanding how more subjective and more objective parts of applicant evaluations play together. Ensuring objective assessment and selection criteria, and relying on objectively observable information cues, has been suggested to boost gender equality, also in academic recruitment (Heilman, 2012; Herschberg et al., 2015). To test for gender bias despite having objectively the exact same information cues given for male vs. female applicants (regarding publication records, showing willingness to cooperate, and having a network recommendation), we explore whether, in a situation in which applicants are comparable, success patterns lead to different selection recommendation for women vs. men.

Third, we show how combining analyses of responses to vignettes and interview questions, as well as think-a-loud comments, helped us to understand the full picture including more subjective and more objective parts of applicant assessments. We follow calls to look “behind the numbers” of quantitative survey ratings (Einola and Alvesson, 2021) showcasing an approach combining qualitative and quantitative data and apply both, inductive coding and fuzzy set qualitative comparative analysis (fsQCA). The logic of QCA fits the data structure of conjoint experiments and helps us understand success patterns that led to a high selection recommendation for men and women. The conjoint experiment indicates gender-independent and -dependent success patterns, which we can contextualize and interpret in light of interviewees’ qualitative statements.

## Theoretical background

Due to general uncertainty in selection decisions and incomplete information about applicants, evaluators use heuristics (Tversky and Kahneman, 1974) and signals (Spence, 1973; Rynes, 1991; Connelly et al., 2011) to assess applicants’ suitability for a specific position. In the following, we first theoretically introduce heuristics, stereotyping, and signaling and subsequently discuss how they play out in academic selection committees.

### Heuristics and stereotyping in recruitment

*Heuristics* explain how judgments are made in situations of uncertainty (Tversky and Kahneman, 1974). For instance, stereotypes are representativeness and similarity heuristics leading to mental “shortcuts” in applicant assessments (Tversky and Kahneman, 1974; Kunda and Thagard, 1996; Heilman, 2012). Heuristics assess the likelihood of an applicant’s success in a specific job is based on the applicant’s similarity to former “typical” successful job holders (Tversky and Kahneman, 1974). Due to gendered success models, requirements are likely to be perceived more stereotypically male the higher the perceived status of the work context and more stereotypically female the higher women’s expected share in the work context (Cejka and Eagly, 1999; Koenig et al., 2011; Dutz et al., 2022). That is, perceived requirements across work contexts are stereotyped.

Furthermore, stereotype-based heuristics account for ascribing different attributes to men vs. women based on their gender (i.e., descriptive gender stereotypes; Heilman, 2001, 2012; Kunda and Thagard, 1996). Generally, men are likely to be ascribed stereotypical male *agency* (e.g., rational, analytical, and ambitious) and women are likely to be ascribed stereotypical female *communality* (e.g., emotional, sensitive, and modest). That way, heuristics account for *other-stereotyping*, as they fuel gender biases in how evaluators judge applicants and their fit to a job (Heilman, 1983, 2012; Hentschel et al., 2019).

Additionally, stereotype-based heuristics fuel *self-stereotyping*. Stereotypical perceptions influence individuals’ self-characterizations based on their gender (Hentschel et al., 2019), and self-assessments of their fit to a gendered work context (Heilman, 1983, 2012). Therefore, women may see themselves as less agentic than men, and as less qualified or suitable for male-typed positions, such as high-status positions, particularly in male-dominated fields. In addition, to avoid social backlash, they may actively withdraw from displaying agentic traits and behaviors, such as self-promotion and power-seeking (Rudman, 1998; Moss-Racusin and Rudman, 2010; Okimoto and Brescoll, 2010).

Thus, heuristics fuel various stereotype-based biases. Most relevant in influencing evaluators’ perceptions in applicant assessments are stereotyped requirements of jobs and stereotypes applied to applicants such as due to their gender. One way to reduce such gender biases is carefully assessing the actually needed (rather than stereotypical) qualifications and skills for the job, and applicants’ respective attributes (Heilman, 2012). When assessment criteria are clear, evaluators can define concrete signals to look for in applicants.

### Signals in recruitment

Especially in situations of incomplete information such as applicant assessments, evaluators rely on *signals* (e.g., applicant details in application materials) to infer attributes which they cannot directly observe (e.g., knowledge, skills, and abilities; Spence, 1973; Rynes, 1991; Connelly et al., 2011). For instance, they include information applicants provide in their CV (e.g., on performance outputs or qualifications for the job) or during the job interview (e.g., on their ability or willingness to work in a team), or information that others provide about applicants (e.g., former employers in reputation letters). Thus, signals serve as information cues for evaluators to form a picture of applicants. The signals evaluators observe and interpret during recruitment help decreasing stereotypical perceptions because signals provide information on the applicants’ qualities overriding what is inferred from their gender. However, the future success of applicants is uncertain and, thus, the overall assessment of an applicant still requires subjective interpretation. Furthermore, although signals help to override stereotypical perceptions of applicants, stereotypes can still bias perceptions (see Nieva and Gutek, 1980; Heilman, 2012).

Although signals can foster objectivity in applicant assessments when assessment criteria (and signals to look for) are clearly defined, based on the actual requirements, and objectively assessable, in practice, the criteria for assessment and selection are often ambiguous, fueling subjectivity and influences of heuristics (Nieva and Gutek, 1980; Heilman and Haynes, 2006; Heilman, 2012). Furthermore, even if a work context promotes clearly defined criteria and objective assessment, as it is in academia, the criteria may still be subjectively interpreted and construed differently for men and women (van den Brink and Benschop, 2012; Herschberg et al., 2015). That is, criteria and relevant signals may still be influenced by gendered success models and may be interpreted or weighted differently for men vs. women. This may be particularly true for high-status positions and male-dominated fields such as STEM professorships.

### Recruitment for STEM professorships

In our research we investigate the mechanisms of heuristics, stereotyping, and signaling specifically in the context of STEM professorships. Professorships are high-status positions and male-dominated, particularly in STEM fields (van den Brink and Benschop, 2014; Carli et al., 2016; Catalyst, 2020c; GWK, 2020). Additionally, academia is a particularly interesting context to analyze gendered arguments despite desired objectivity because of high efforts for clearly defined and objectively assessable criteria, while discussions in selection committees are still gendered (e.g., van den Brink and Benschop, 2012; Herschberg et al., 2015).

#### Heuristics and stereotypes in recruitment for STEM professorships

Prior research provides evidence for the influence of heuristics and stereotypes in recruitment for STEM professorships. The high-status leadership positions in male-dominated fields possess a clear male stereotype influencing perceived job requirements (Cejka and Eagly, 1999; Koenig et al., 2011; Heilman, 2012; Dutz et al., 2022). A male stereotyped success model in academia is further reflected in the male-typed construction of academic “excellence,” referring to scientific competence, which is – although a halo selection criterion – ambiguously defined and inherently gendered (van den Brink and Benschop, 2012). Therefore, the importance of stereotypical male applicant attributes is likely overestimated in assessments. Moreover, to “preserve” the gendered success model evaluators take into account “physical appearance, self-presentation, and perceived personality and leadership potential as valid criteria that can overrule other, more formally specified criteria” (van den Brink and Benschop, 2012, p. 9).

#### Applicant signals in recruitment for STEM professorships

One unquestionable assessment criterion in recruitment for professorships is the publication track record (see, e.g., Bedeian, 2004). Nevertheless, the actual requirements are more diverse (e.g., Eagly and Carli, 2003; Braun et al., 2013; Rehbock et al., 2021). For example, scientific output such as publications are most often team efforts; that is, cooperativeness, a stereotypical female quality (Heilman, 2012), is most likely an integral part of past and future achievements (Rehbock et al., 2021). Moreover, visibility and a good reputation in the scientific network likely help applicants (van den Brink and Benschop, 2014), while networking covers stereotypical male (e.g., impression management; Rudman, 1998) as well as stereotypical female aspects (e.g., interpersonal skills; Heilman, 2012; Gazdag et al., 2022). Thus, core evaluation criteria likely include publications, the willingness to cooperate, and having a strong network. Importantly, those criteria, despite being potentially gendered somewhat intangible, can be pre-defined and respective signals can be explicitly expressed by/for one applicant but not by/for another.

*Publications* are usually a crucial and formalized selection criterion (Herschberg et al., 2015). They signal scientific competence, and therefore – based on quality indicators such as journal impact factors and citations and on quantity – can be an indicator of research success that is objectively assessable. Although evaluating publications may also entail subjective elements (e.g., evaluating publications by reading them; Herschberg et al., 2015), decisions on applicants likely get more complicated – and more subjective – when applicants cannot be clearly distinguished by looking at their publications. When anticipating applicants’ (future) research success and thereby their potential (e.g., for more junior researchers), due to heuristics and stereotypes it is likely that evaluators underrate the potential of minority applicants (e.g., women in STEM; Norton et al., 2004; Uhlmann and Cohen, 2005; Heilman, 2012; van den Brink and Benschop, 2012).

Additionally, *willingness to cooperate*, in general or on specific research projects, is likely crucial (Rehbock et al., 2021). While in practice difficult to assess and anticipate in applicants, applicants can emphasize their willingness to cooperate during the recruitment process. Applicants can express their willingness to cooperate by showing that they are informed and intend engagement with prospective faculty colleagues in the hiring university. Furthermore, cooperativeness as a core competency for generating scientific output in teams is likely seen as beneficial in view of future shared achievements as well as shared responsibilities among faculty members, such as academic administration tasks (Herschberg et al., 2015; Rehbock et al., 2021). Thus, cooperativeness can also be judged from how applicants describe their previous collaborations.

Furthermore, *network recommendations* can be beneficial for applicants. Network effects include higher visibility and reputation due to being part of a powerful network, which is a desirable characteristic in applicants (van den Brink and Benschop, 2014; Herschberg et al., 2015). Particularly in academia, “gatekeepers” dominate professional networks and make recruitment decisions (e.g., professors or deans, most often male), having a lot of influence and “the power of inclusion and exclusion” (van den Brink and Benschop, 2014; p. 1). Women are underrepresented in these networks and, therefore, less visible (van den Brink and Benschop, 2014). Nevertheless, whether or not someone in the applicant’s network expressed a recommendation for the applicant can be observed objectively.

Importantly, in STEM networks and also in STEM departments, there is a “chilly climate” for women (Hinsley et al., 2017; Casad et al., 2021). It is more difficult for women to operate in these contexts, due to stereotype biases (e.g., being perceived as “undeserving”; McKinnon and O’Connell, 2020), sexism, and structures mostly made for men (e.g., in regards to (low) family or care related support; Greider et al., 2019; Casad et al., 2021). Moreover, gatekeepers seem to reason that social interactions are more complicated with women in “manly” work climates (van den Brink and Benschop, 2014), using chilly climate arguments to “protect” women from entering the field or higher positions, rather than making efforts to climate or culture change and successful inclusion (see, e.g., Roberson, 2006; Mor Barak, 2015). Although this may be meant well for women, it often is an additional barrier.

Concluding, there are both subjective heuristics and objectively observable signals influencing evaluators’ perceptions and discussion of applicants for STEM professorships. We investigate how heuristics and stereotyping contribute to the persistence of gendered arguments in applicant assessments in these contexts illuminating the duality of subjective interpretation of seemingly objective criteria. We further test for gender bias despite having objectively the same information cues for male and female applicants (regarding publication records, showing willingness to cooperate, and having a network recommendation); we explore whether, in a situation in which applicants are comparable, success patterns are gendered, i.e., whether signals lead to different selection recommendation for men and women.

## Materials and methods

To investigate gendered arguments and gendered success patterns in applicant assessments for STEM professorships, we implemented a mixed methods approach including 45 qualitative interviews that incorporated a conjoint experiment. The interviews comprised open-ended questions that we analyzed qualitatively as well as reactions to vignettes that we analyzed qualitatively and quantitatively. The interviewees quantitatively rated hypothetical applicant profiles, while thinking aloud, commenting on their evaluations and thoughts behind their ratings (see also Einola and Alvesson, 2021). The vignettes were introduced to stimulate their thoughts on specific applicant profiles and to investigate evaluations of male vs. female applicants based on comparable applicant profiles. Capturing interviewees’ answers to our questions and their evaluations of hypothetical applicants, we could analyze gendered arguments in appointment committees. Additionally, based on quantitative ratings of hypothetical applicants, we could examine success patterns for male vs. female applicants.

### Research context

Our research context, i.e., the German academic system, is characterized by a lack of permanent positions, posing particular challenges for young scientists (Brechelmacher et al., 2015). For instance in 2020, there were 49,293 professors in Germany (Destatis, 2021). Per year, about 30,000 PhD students are graduating and *ca*. 33%, and another undecided *ca*. 35%, are potentially striving for a professorship that becomes vacant (Nacaps, 2021), e.g., due to professors retiring (in 2021 *ca*. 2,6%; Destatis, 2021; Zeitler, 2021). An approximate calculation of the probability of PhDs becoming professors results in 7% in mathematics/natural sciences and 20% in engineering (Krempkow, 2017). Illustrating the career time span, the average age at PhD completion was about 30 in 2020 (Destatis, 2021), and the average age of being appointed to a permanent position is still above 40 (KBWN, 2021; Zeitler, 2021). Full professors typically hold permanent positions, most often holding an own “chair” including leadership responsibility (Muller-Camen and Salzgeber, 2005; Braun et al., 2013). Due to the far-reaching nature of lifetime appointment, appointment decisions are “high-risk decisions” under uncertainty (Tversky and Kahneman, 1974; van den Brink and Benschop, 2014).

### Research sample

The decisions on professorial appointments are made in appointment committees. Professors regularly take part and lead the discussions on applicants in those committees (van den Brink and Benschop, 2014; Frey et al., 2015). In their disciplines, they are “gatekeepers” in recruitment for professors-to-be and of respective academic (social and career) networks (van den Brink and Benschop, 2014). We applied purposive sampling (Patton, 2002) and intended to recruit interview partners able to share rich information on discussions in appointment committees for STEM professorships. Thus, we recruited tenured STEM professors attending appointment committees as our interviewees, balancing their mean age to avoid age bias. We recruited the professors *via* e-mail, asking them for a 30–45-min-interview on success factors of academic careers in STEM disciplines. The interviews were taken over the phone, and the interviewees could fill out the anonymized conjoint experiment survey online. Once new interviews did not lead to the identification of new major themes, we concluded sampling, based on principles of theoretical saturation (Strauss and Corbin, 1998; Gioia et al., 2013).

Finally, we included 45 tenured professors across different STEM disciplines and across different universities all over Germany. Although these positions overall are male-dominated (women currently make up for about 20% of job holders; GWK, 2020), we aimed to interview a similar number of male and female professors (51% female, *M*_age_ = 46.4 years), to account for both perspectives on applicant evaluations and discussions in appointment committees. Table 1 presents participant demographics, whereby the interview partners are presented in different order (arranged by gender) than in-text to guarantee anonymity.

**Table 1:** Sample description.

Gender	STEM discipline
Male	Informatics
Male	Informatics
Male	Electrical engineering
Male	Informatics
Male	Mathematics
Male	Physics
Male	Mathematics
Male	Physics
Male	Physics
Male	Physics
Male	Informatics
Male	Electrical engineering
Male	Informatics
Male	Physics
Male	Electrical engineering
Male	N/a
Male	Mathematics
Male	Physics
Male	Informatics/mathematics
Male	Mathematics
Male	Mathematics
Male	Mathematics
Female	Physics
Female	Informatics
Female	Mechanical engineering
Female	Mathematics
Female	Mathematics
Female	Informatics
Female	N/a
Female	Product engineering
Female	Georesources
Female	Informatics
Female	Mechanical engineering
Female	Physics
Female	Mathematics
Female	Mathematics
Female	Mathematics
Female	Mathematics
Female	Sustainability
Female	Mathematics
Female	Informatics
Female	Electrical engineering
Female	Physics
Female	Physics
Female	Physics

Participants were 45 STEM professors in Germany. N/a indicates that participants did not want to indicate their discipline for the statistic.

### Research design and procedure

#### Interview guideline and questions

We conducted semi-structured interviews based on an interview guideline with pre-defined questions, while allowing to flexibly adapt to the individual conversations (e.g., by asking follow-up questions; see Myers, 2020). The first part of the guideline covered general questions on success factors and barriers for professorial applicants in STEM (e.g., “In your opinion, what are the three most important success factors for being appointed as a professor in your field?”). Then, we presented the interviewees with vignettes showing hypothetical male and female applicants which they evaluated, (1) *qualitatively* by commenting on their evaluation of applicants and (2) *quantitatively* by rating them with respect to selection recommendation (details below). Subsequently, proceeding with the interview guideline, the interviewees were asked more specific open-ended questions on success factors and barriers specifically for female applicants as well as on how gender, other demographics (e.g., age), and family obligations are a matter of discussion in appointment committees (e.g., “Do you see specific success factors for women to be appointed as a professor in your field?” and “How was gender a matter of discussion in appointment committees you were part of?”).

#### Vignettes and conjoint experiment

The vignettes depicting hypothetical applicant profiles construed an assessment scenario, that is, a hypothetical scenario of an appointment committee for the selection of a STEM professor. The interviewees were asked to imagine to be part of the appointment committee (as they have been in “real-world” appointment committees). Sixteen different vignettes represented 16 profiles of “shortlisted” applicants.

The 16 applicant profiles enabled a metric conjoint experiment with multiple applicant attributes varying across profiles. On the one hand, the profiles stimulated the interviewees’ thoughts on applicants and the different attributes (“think aloud” evaluation; Einola and Alvesson, 2021). On the other hand, this setup tested the attributes’ influence on interviewees’ selection recommendation, which they indicated by a quantitative rating. Conjoint experiments are particularly useful to model (assessment) decisions (Domurath and Patzelt, 2016; Warnick et al., 2018). They allow to test for the influence of several attributes simultaneously especially regarding attribute combinations thereby exceeding the explanatory power of traditional experiments. Varied attributes, in our case of construed applicant profiles, present the independent variables in conjoint experiments, while the (quantitative) assessments, in our case the selection recommendation for the applicants, comprise the dependent variable (Domurath and Patzelt, 2016; see also Green et al., 2001). In the applicant profiles, we varied four attributes with two levels each in a fully-crossed within-design (2^4^ = 2 × 2 × 2 × 2 = 16 different combinations, i.e., profiles). Interviewees assessed all possible combinations of attributes in applicant profiles. The varied attributes and their levels were publications (solid vs. outstanding), willingness to cooperate (low vs. high), network recommendation (non-present vs. present), and applicant gender (male vs. female).

Publications, meaning the quality and quantity of applicants’ publications, was included as the most explicit factor or criterion representing scientific competence and amenable for objective assessment (while there are still varying and biased arguments of how and which publications are taken as cues for scientific competence; see, e.g., van den Brink and Benschop, 2012). The vignettes either stated that the applicant has *solid* publications (i.e., meeting but not exceeding average expectations) or *outstanding* publications (i.e., exceeding average expectations).

Willingness to cooperate, meaning applicants’ signaled interest in cooperating with prospective faculty or university colleagues, was included as the second relevant factor in appointment decisions, although less suitable for objective assessment (see, e.g., van den Brink and Benschop, 2012, 2014). Without relevant and unbiased “proof” (such as knowledge of previous collaborations), this may be a factor subjectively discussed in appointment committees, based on impressions of applicants, their conveyed personality, interests, and appearance in formal (and informal) talks. Levels were *low* or *high* willingness to cooperate.

Network recommendation, meaning that the applicant is recommended in some committee member’s personal (research) network, was another potential success factor in our analysis. Network recommendations play a crucial role in appointment decisions, informally discussed rather than as formal criteria, and are likely more common for male than female applicants, especially in STEM fields (van den Brink and Benschop, 2012, 2014). Levels we compared were *non-present* (i.e., there is no present/known recommendation for the applicant) and *present* (i.e., there was present/shared a positive network recommendation for the applicant).

Applicant gender was varied assessing whether thoughts and evaluations differed based on applicant gender. The levels were *male* and *female* indicated by a blurred image/icon of a male or female face and the indication of Mr. or Ms. in the profiles.

In their assessments, the interviewees were to assume that the applicants essentially differ in regards to the four varied attributes on the profiles. In the interviews, we assessed whether publications, willingness to cooperate, and network recommendation were seen as important and realistic selection criteria (and, thus, reasonable to be selected as conjoint experiment decision criteria; see Warnick et al., 2018). The attributes were evaluated realistic and important selection criteria, whereby publications and willingness to cooperate where often seen as “showstopper” criteria [e.g., Interview (Int.) 26(male)] and network recommendations were evaluated more controversially. A few interviewees highlighted that the latter should not be influencing selection decisions, while others emphasized that those (still) have crucial influence in discussions about applicants, especially when applicants’ documents do not allow for clear judgment of their suitability (e.g., Int. 10(female)).

To obtain a quantitative measure of the dependent variable, the participants indicated for each applicant profile their selection recommendation, on a 1-item 7-point Likert scale (“Please indicate based on the profile information: Would you advocate for this person to be selected for the list of applicants that are considered further?”; 1, “no, definitely not” to 7, “yes, definitely”), modified from Heilman and Okimoto (2008). To test for test–retest reliability of the interviewees’ quantitative ratings on this measure, we randomly replicated four out of the 16 profiles on each participant (Warnick et al., 2018). We also included a practice profile to familiarize participants with the setting (such that, in total, the interviewees rated 21 profiles; Warnick et al., 2018). Both, the replication profiles as well as practice profile, were not included in the main analysis (Aiman-Smith et al., 2002; Warnick et al., 2018). The conjoint experiment survey ended with a demographic survey.

### Data analysis

#### Inductive coding and visualization of emerging themes

We analyzed the qualitative data of the STEM professors (i.e., their verbal answers to open-ended questions and verbal statements on hypothetical applicant profiles) by means of qualitative, inductive coding (Gioia et al., 2013; Eisenhardt et al., 2016). In addition, in our data analysis process, we discussed different visualizations of the emerging themes (Miles and Huberman, 1994).

Following the inductive analysis procedure suggested by Gioia et al. (2013), we initially applied a *first order analysis* of categories arising from the data (see also Strauss and Corbin, 1998). Thus, we initially closely adhered to the subjects’ verbal statements and not yet formed (theoretical) higher-order categories, allowing a large number of parallel first order categories to emerge from the analysis. Those codes captured statements in which participants outlined their view on how appointment committees treat women differently than men, which behaviors they observed in male and female applicants, and how they evaluate participants regarding specific criteria including publications, cooperation, and networks.

In a second step, we applied a *second order analysis* looking for similarities and differences between the initial categories. Thereby, we identified “whether the emerging themes suggest concepts that might help us describe and explain the phenomena we are observing” (Gioia et al., 2013, p. 20). In doing so, we went back and forth between data-based codes and theoretical level themes. We focused on those emerging themes that are of particular interest as they offer new theoretical insights (Corley and Gioia, 2004; Gioia et al., 2013). For example, on themes that indicate how women are perceived as “extraordinary” or particularly salient questioning of female applicants in appointment committee discussions.

Third, we further distilled our second order themes to *aggregate dimensions* towards a more abstract and interpretative, theoretical view (Gioia et al., 2013). In the findings section, we present our data structure, explaining how we went from the original data to our theoretically aggregated dimensions (Corley and Gioia, 2004; Gioia et al., 2013). For example, we grouped codes referring to perceived self-questioning of women and those referring to evaluators questioning similar attributes in women (abilities, general traits, commitment).

#### FsQCA of quantitative data

To analyze STEM professors’ quantitative ratings (i.e., the quantitative scores they indicated for the different vignettes of hypothetical applicants), we performed a fuzzy-set qualitative comparative analysis (fsQCA; Ragin, 2008a).

The logic of QCA is to identify combinations or *configurations* of factors that indicate a specific outcome of interest (Ragin, 2008b; Pappas and Woodside, 2021), whereby the analysis allows to capture “multiple paths that lead to the same outcome” (Pappas et al., 2020; p. 5). Therefore, applying QCA, we used a configurational approach (Delery and Doty, 1996; Fiss, 2007), investigating configurations of theory-based, manipulated attributes (their presence or absence, respectively) that lead to a specific outcome, rather than a variance-based approach focusing on attributes’ isolated effects (Pappas and Woodside, 2021). As our outcome variable was measured on a 7-point Likert scale (i.e., non-binary), we performed a particular type of QCA, i.e., fuzzy-set QCA (Ragin, 2008b; Pappas and Woodside, 2021). Enabling higher complexity of variable levels (Ragin, 2000; Rihoux and Ragin, 2008), fsQCA is a popular variation of QCA to analyze quantitative data (Pappas and Woodside, 2021), increasingly used in business and management (Kumar et al., 2022), entrepreneurship, and innovation research (Kraus et al., 2018).

We used fsQCA to analyze which configurations of the manipulated attributes on hypothetical applicant profiles led to a high selection recommendation, including applicant gender and, additionally, evaluator (i.e., interviewee) gender in configurations. To do so, we analyzed all possible combinations of factors to predict the outcome of high compared to low selection recommendations (Woodside, 2013, 2016; Pappas and Woodside, 2021) based on fsQCA software (3rd version; Ragin and Davey, 2016).

First, we employed *data calibration* (Ragin, 2008a; Pappas and Woodside, 2021) converting our non-binary outcome variable “selection recommendation” into degrees of membership of 0 to 1 by setting three anchor points: full membership, crossover point, and full non-membership. Full membership referred to “high selection recommendation” and full non-membership to “no high selection recommendation” or “low selection recommendation”; the cross-over point indicated “the value where there is maximum ambiguity as to whether a case is more in or more out of the target set” (Pappas and Woodside, 2021; p. 8). The three anchor points were set at 6, 4, and 2, as suggested for 7-point Likert scales (Ordanini et al., 2014; Pappas et al., 2016).^1^ We did not calibrate the variables for the manipulated attributes on hypothetical applicant profiles, nor evaluator gender, due to their already binary levels.

Second, we generated a *truth table* (Ragin, 2008a; Pappas and Woodside, 2021). The truth table in fsQCA displays all possible configurations of factors (in our case of the manipulated attributes in the conjoint experiment design, i.e., publications, willingness to cooperate, network recommendation, applicant gender; as well as evaluator gender). As the attributes and evaluator gender had two levels each, the truth table showed 32 different configurations (2^5^ = 32 combinations). The truth table shows the frequency for each possible configuration (Pappas and Woodside, 2021). As in our conjoint experiment every participant rated all vignettes/applicant profiles, thereby all possible configurations of attribute levels, the frequency for each configuration was largely fixed by the number of participants; only the frequency for configurations with evaluator gender varied as we had 23 female and 22 male participants.^2^ The truth table also shows the configurations’ consistency, where we set the recommended thresholds for “raw consistency” at minimum 0.75 (Rihoux and Ragin, 2008) and for “proportional reduction in inconsistency” at 0.7 (Greckhamer et al., 2018; Pappas and Woodside, 2021).

In a third step, we proceeded with *obtaining the configurations* (or “solutions”), setting the individual factors as being “present or absent” in the configurations (Ragin, 2008a; Pappas and Woodside, 2021). For high selection recommendations, the analysis yielded three different configurations; for low selection recommendations, the analysis yielded two different configurations. We further examined what are core or peripheral conditions in the obtained configurations by comparing the intermediate and parsimonious solutions identified (Pappas and Woodside, 2021; Fiss, 2011).

## Empirical findings

### Qualitative data

In the following, we present the results of our inductive coding of STEM professors’ qualitative, verbatim statements to open-ended questions and hypothetical applicant profiles. The abbreviations “m” and “f” indicate the gender of interview partners as male or female.

#### General subjectivity in applicant assessments

Interview statements highlighted the general subjectivity in discussions of applicants in appointment committees. As explained by one of the professors (Int. 20 (m)), “[while] appointment processes are quite objectivized on paper, in reality, they develop their own dynamics that do not necessarily make them as objective as they seem.” Other professors emphasized their perception of “subjectivity” in applicant discussions (e.g., Int. 27(m), 29(f)). One interviewee (10, f) specified that “occasionally, when the documents do not allow definite judgment, it only takes a few words to suddenly push a certain applicant.” While the professors mentioned some explicit criteria for assessment and selection (such as publications, e.g., Int. 9(m), 13(m), 20(m), 26(m), 44(f)), they repeatedly highlighted that discussions in appointment committees often gain momentum in unpredictable directions (also Int. 13(m), 15(m), 19(f), 37(m)).

#### Gendered arguments in applicant assessments

**Figure 1:**
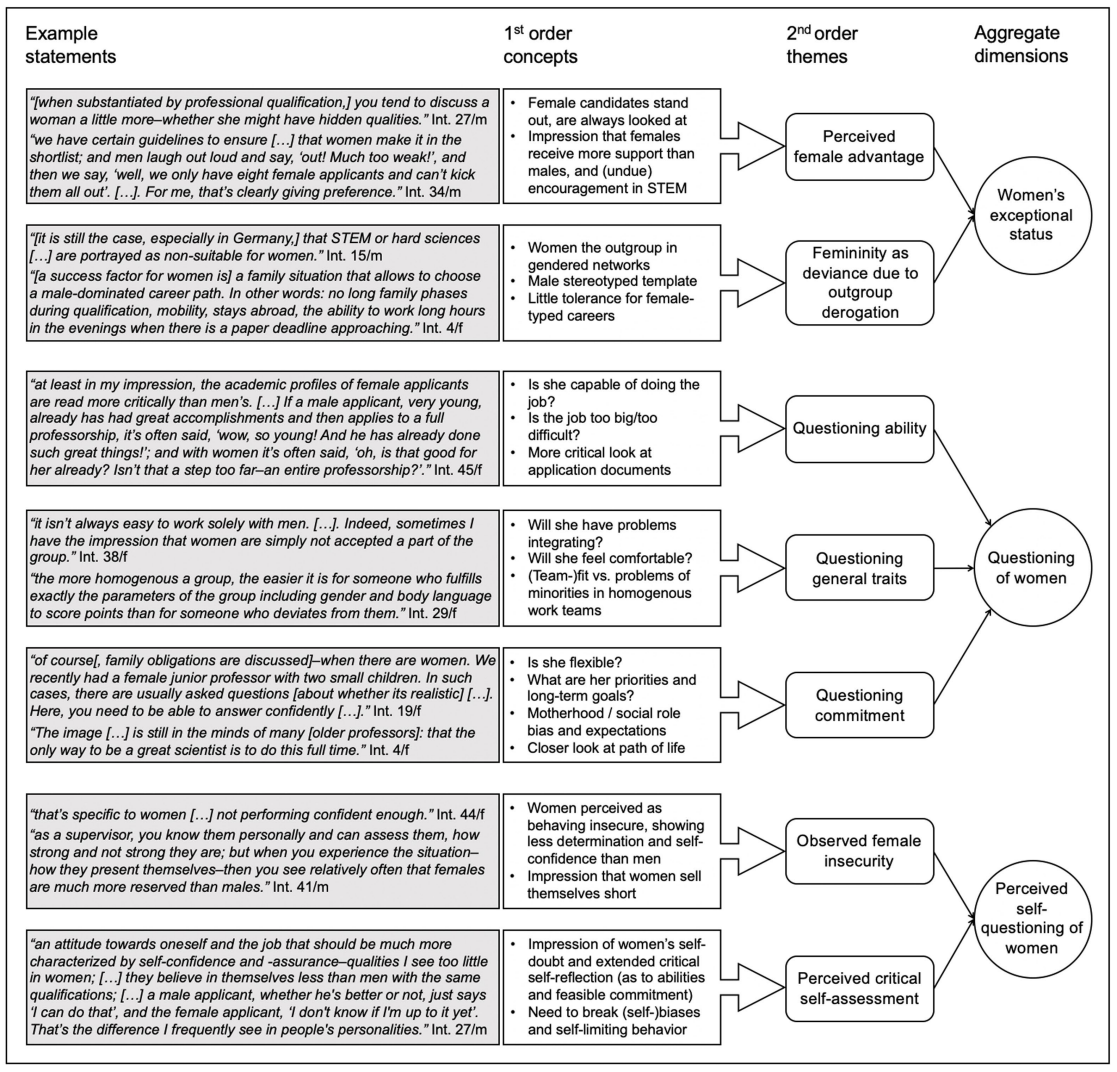
Data structure of inductive coding of STEM professors’ qualitative, verbatim statements [according to Gioia et al. (2013)]. “m” and “f” indicate the gender of interview partners as male or female.

Interview statements further highlighted that applicants’ gender is an issue explicitly or implicitly in appointment committee discussions, reflected in different types of gendered arguments (Figure 1).

##### Women’s exceptional status

Among the professors there seemed to be a consensus that women as applicants for STEM professorships have an exceptional status; however, while some professors were arguing for a *perceived female advantage* due to women’s exceptional status, other statements pointed to *femininity as deviance due to outgroup derogation*.

###### Perceived female advantage

Some professors described an “advantage” of female applicants standing out due to their gender, arguing that, in appointment committees, female applicants’ profiles are always looked at and discussed in more detail. For example, Interviewee 24(m) stated:

“You can be pretty sure to not be overlooked [as a female applicant for a STEM professorship]; you still need to be good, but if you are, you don’t need much luck […]. If this is reflected in two objective criteria: publications and visibility–that you are perceived to be good–then, you almost surely find a suitable position.”

More generally, Interviewee 1(m) argued that women in STEM enjoy more support and encouragement than men in STEM such as when describing that “everyone wants women to do technical staff […]; and they literally bring out the red carpet for women.” Interviewee 27(m) sees “women are privileged [in appointment decisions] but only to certain extent–when substantiated by professional qualification.” Interestingly however, some interviewees say that even if they prefer women, sometimes they have difficulties to argue for them, such as Interviewee 12(m): “There are always a lot of top candidates–and I am preferring females; but the majority would prefer males and that’s why there are hardly any women.” Other interviewees perceive the female advantage even clearer, for example Interviewee 34(m) stated “with the same criteria met, female applicants are preferred.” Thus, interview statements indicate that women might have an advantage in some situations.

###### Femininity as deviance due to outgroup derogation

Other interview statements rather point to femininity as deviance to a male stereotyped template of selecting applicants. “You have a problem if you don’t fit the frame–brutally speaking, somehow, if you are not the big alpha male,” explained one of the professors (Int. 12(m)). Another professor (Int. 4(f)) described: “Specific types of CVs are favored–which are statistically more found in men,” and Interviewee 29(f) specified that there is little tolerance for female-typed careers:

“The actual understanding of the situation and of careers that are not perfectly linear and do not follow the typical pattern of a male career but may have interruptions or deviations–for such unusual biographies, there is no high tolerance in committees.”

In line with this logic were arguments assigning women an outgroup status, for example in professional networks. For instance, Interviewee 43(m) argued, “I can imagine–since men still dominate the field–that it might be easier for them to be recommended within their network.” Another professor (Int. 37(m)) explained “a lot of socializing at workshops or conferences takes place over a beer in the evenings–at this point, women are most often already gone.” Thus, being female is seen as deviance, and women are perceived to be less likely integrated in the network, such that they often end up as “outgroup.”

##### Questioning of women

In addition to arguments with respect to women’s exceptional status, we observed that there was a lot of questioning of female applicants in the statements of our interviewees. We observed three kinds of questioning categories, i.e., questioning ability, general traits, and commitment.

###### Questioning ability

Statements of interviewees pointed to questioning of women’s ability. Outlining that the ability is questioned, one of the professors (Int. 45(f)) described:

“With women it is often assumed that a job might be too big for them, too difficult, too early. This shows that gender is discussed. It is not discussed in a gender-neutral way, although we all claim for ourselves that we are super neutral and not biased; that is simply not true.”

Another professor (Int. 35(f)) framed it differently: “My impression is often that women have to be slightly better than comparable men; because women are taken a more critical look at.” Both statements point to preconceived notions of female applicants’ potential insufficient ability, respectively to (prior) skepticism of whether female applicants are able to handle the job of a STEM professor. Interviewee 14(m) reported questioning in a more explicit form: “In engineering commissions, I often witnessed openly expressed bias: Can *women* even do the job?,” while referring to reactions of older committee members in particular. Additionally, interviewee statements reflect stereotypical thinking patterns in abilities needed:

“Some soft skills may not be valued sufficiently–because factors such as ‘women’s groups work better and women create a better group atmosphere’, what you hear often, are hardly considered in appointments […]. In this regard, I can imagine that certain qualities of women are undervalued.” (Int. 24(m))

Another professor (Int. 13(m)) emphasized the need of “trying to get away from STEM being somehow more masculine and social professions more feminine,” connecting to gender-stereotypical ability requirements across (male- vs. female-dominated) domains. Thus, we could observe salient (though often implicit) scrutinizing of female applicants’ (general) ability for the jobs, including references that a more critical look is taken at their ability.

###### Questioning general traits

The professors’ statements also pointed to questioning of whether women’s traits fit the male-dominated environments and groups. Under this category, we summarized statements referring to considerations of (team-)fit (as female minority and “rare bird” among male colleagues; Int. 6(m)), and whether women will have problems integrating or will feel uncomfortable due to (women’s vs. men’s) alleged different character and behavioral traits and style of interacting. For instance, more generally, Interviewee 6(m) stated that “if you are in a group of 20 scientific staff members and the only women, this also has an influence on the whole group dynamic, of course.” The professor further explained: “A women alone in a male-dominated group; that can cause difficulties in terms of assertiveness, discourse, etc.–there are studies on that.” Another professor stated:

“The conversational atmosphere, the way men interact–that’s different from how women interact […]; you have to be able to adjust to it. You must not react too sensitive to mocking remarks or dirty jokes […]. I feel like those [women] who made it are resilient in that regard.” (Int. 19(f))

More so, Interviewee 9(m) described the impression that “in a subtle, subjective way, appointment committees tend to appoint people who fit the majority of the people in the commission best.” While “the conceit of what is a professor is still very present” (Int. 12(m)), women seem to be perceived as different. All those statements indicated that the fit of women regarding their general traits is questioned.

###### Questioning commitment

Besides questioning female applicants’ ability and general traits, we found questioning of their commitment, flexibility, and whether they may have other priorities than or besides the job, while the job is assumed to require the job holder’s full dedication. One of the professors explained more detailed (Int. 6(m)):

“You are usually looking for someone who can spend a lot of time [on the job] and, of course, you don’t want to put someone in the situation where he or she is overchallenged when appointed. Thus, you try to find out very precisely what the candidate’s life organization looks like–even if, of course, this should not really be influencing the decision […]. However, when it comes to figuring out how likely the candidate is to accept the position and to do the job the way you think it should be done–these are rather soft criteria you cannot easily quantify. Then it is also discussed: What is the family situation like? What is the person’s goal in life–generally speaking? Does it fit a science career?”

Another professor (Int. 18(f)) exemplified in regards to a female applicant: “Certainly, you think about whether they’ll really come here: Once we also had a young woman with two children–single–so about how she wants to solve that.” Interviewee 9(m) further explained that, in women, “especially when it comes to children, the self-confident appearance must be real; if just saying ‘Yes–I will manage that’ but not believing so, people will notice that.” This category of statements also reflected traditional role expectations or fulfillment, generally existing or anticipated. For instance, one professor (Int. 31(f)) stated, “it’s [still] rather that women are centered on their husband and their husband’s careers than the other way around […]; I personally know few female colleagues where it was the case that their husbands oriented themselves to what their wife needs, obtained, or has to do [career-wise].” These and similar statements indicated the questioning of women’s (general) commitment to the jobs, in regards to time and work investment.

##### Perceived self-questioning of women

Analyzing and aggregating the emerging themes related to gender, we further found interviewed professors reported they *observed female insecurity* in appointment processes or *perceive critical self-assessment*, particularly in women.

###### Observed female insecurity

This category outlines statements concerned with women’s demeanor and appearance in appointment committees, specifically summarizing professors’ observations or perceptions of women behaving insecure, showing less self-confidence or determination than men, in the process. One professor described:

“It can still be observed that women behave needlessly modest and insecure. I think men often have the habit of being more self-confident, perhaps too self-confident, and women sometimes sell themselves short.” (Int. 26(m))

Other professors share the observation of low or less self-confident demeanor and presentation of their achievements among women, in appointment committees (e.g., Int. 19(f), 44(f)). More directed towards showing determination in appointment committees and when describing their vision and future professional plans, one of the interviewees (Int. 23(f)) explained:

“They [the applicants] need to have a plan of how they want to fill their subject in the future–I think, in many women, this is the biggest weakness. They signal a lot of cooperativeness; but when asked ‘what do you want to do in this position in the future?’, many of them have a very vague idea. Far less clear than men often do.”

These observations or impressions were shared in the sense of being perceived as barriers to women’s chances in appointment processes and their career advancement in general; or, in a similar fashion, as recommendations for female applicants in particular. For instance, Interviewee 3(f) recommended:

“[I would recommend to women] to not be shy–somehow, to assert yourself a bit and clearly state: ‘I did that’; because often I have the feeling that women do that less often. For example, when there is a project that was done in collaboration with others, to clearly say: ‘Yes, I did that’; because I think women tend to take a back seat.”

Thus, especially in female applicants, professors seem to observe insecurity in their behavior and appearance, when they are presenting themselves to be appointed as professors.

###### Perceived critical self-assessment

Another theme pointing to perceived self-questioning of women emerging from the data was perceived critical self-assessment, particularly in women. In this category, we clustered statements that point to a general impression of women in STEM academia having high levels of self-doubt and engaging in extended critical self-reflection; thus, outlining a general bias participating professors see in women’s perception or belief about themselves and their own abilities. For instance, Interviewee 41(m) described the following impression:

“The average female candidate on her career path has more self-doubt and self-criticism, and also expresses them openly. While the typical ‘male STEM’ is a star–at least he thinks he is.”

Another professor (Int. 19(f)) described that self-questioning is even a good feature in science careers but at the same time one needs to be self-assured when pursuing a professorship:

“Women are more covered in doubt. In science, I believe self-questioning is even a good feature–but pursuing a professorship […] a self-assured manner is key to be seen and perceived as positive and successful.”

Additionally, interviewees described the situation of pursuing a STEM professorship in Germany. They emphasized high uncertainty because of non-permanent contracts and high mobility demands including regular changes of location until appointed as a professor as potentially arousing more doubt in female than male scientists in striving to reconcile work and family (e.g., Int. 20(m), 24(m), 31(f), 33(f), 34(m), 35(f)). All those statements indicated that professors see a particularly critical self-assessment, including high self-doubt, as a barrier in female scientists and applicants for STEM professorships.

#### Interplay of gendered arguments

**Figure 2:**
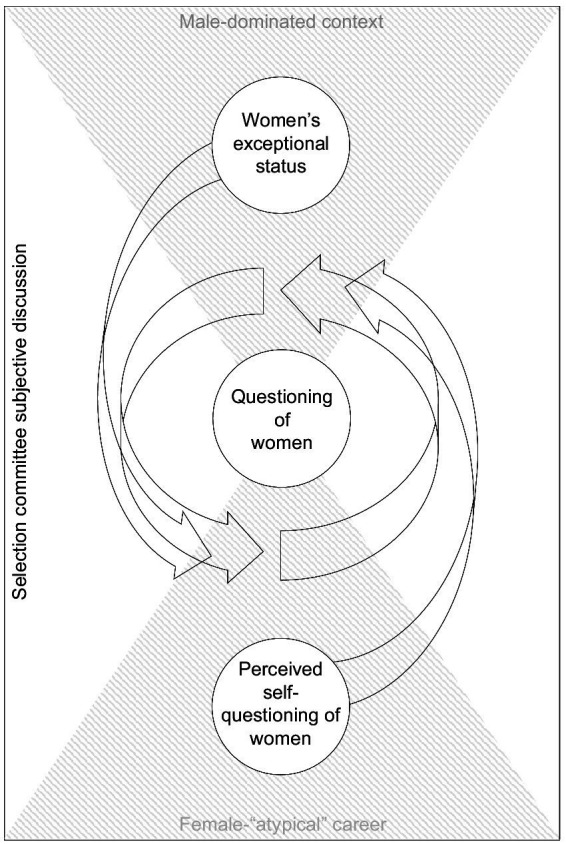
Selection committee subjective discussion of female applicants for STEM professorships (model based on 45 interviews with STEM professors in Germany).

In discussions of applicants the gendered arguments are unlikely to be clearly separated (e.g., general traits may be interpreted as abilities) and reciprocally bias arguments. Building on our findings, Figure 2 illustrates how the male-dominated context (triangle on the top) and the gender “atypical” career of female applicants (triangle on the bottom) shape women’s exceptional status and the perceived self-questioning of women which may in turn fuel the questioning of women (arrows towards the questioning circle) stemming from questioning their fit regarding ability, general traits, and/or commitment.

### Quantitative data

Besides analyzing STEM professors’ qualitative verbatim statements, we explored their quantitative ratings of hypothetical applicants in a fsQCA. We explored how combinations of different applicant success factors (their publications, willingness to cooperate, and network recommendation) and demographics (applicant gender as well as evaluator, i.e., interviewee, gender) led to high vs. low selection recommendations for hypothetical applicants.

We found three configurations (consistency cutoff at 0.755) associated with *high selection recommendations*, presented in Table 2. Configuration 1 of the present core conditions *outstanding* publications and *high* willingness to cooperate led to high selection recommendations, regardless of whether there was a network recommendation, applicant gender, and evaluator gender. Configuration 1 was the configuration associated with high selection recommendations with the highest consistency and coverage. Additionally, configuration 2 of the present core conditions of a *female applicant*, a *high* willingness to cooperate, and a *present* network recommendation led to high selection recommendations, and then whether publications were solid or outstanding and evaluator gender did not matter. Moreover, configuration 3 of the present core conditions *female evaluator*, a *high* willingness to cooperate, and a *present* network recommendation led to high selection recommendations, and here applicant gender and whether the applicant had solid or outstanding publications did not matter. Accordingly, we found one configuration that led to high selection recommendations independent of gender (configuration 1), one configuration that requires female applicant gender (configuration 2), and one configuration that requires female evaluator gender (configuration 3). This indicates that publications and the willingness to cooperate are general success factors, a network recommendation may become important for female applicants, and female evaluators seem to care about a network recommendation.

**Table 2:** Configurations leading to high selection recommendation.

	Configuration		
	1	2	3
Success factors			
Publications	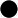		
Willingness to cooperate	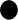	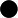	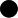
Network recommendation		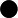	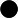
Demographics			
Applicant gender		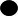	
Evaluator gender			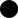
Consistency	0.976	0.878	0.878
Raw coverage	0.414	0.186	0.191
Unique coverage	0.256	0.039	0.041
Overall consistency: 0.918			
Overall coverage: 0.537			

Black circles indicate the presence of a condition (i.e., publications *outstanding*, willingness to cooperate *high*, network recommendation *present*, applicant gender *female*, and/or evaluator gender *female*). All conditions are core conditions. Blank space indicates a “don’t care” condition.

We found two configurations (consistency cutoff at 0.892) associated with *low selection recommendations*, presented in Table 3. The configurations indicate that applicants received low selection recommendations when some core conditions were *absent*. The first configuration (configuration 1 in Table 3) showed that, with a *male* *evaluator*, *solid* (i.e., not outstanding) publications and *low* (i.e., not high) willingness to cooperate led to low selection recommendations, regardless of whether there was a network recommendation and of applicant gender. The second configuration (configuration 2 in Table 3), with similar consistency and coverage, showed a combination of *solid* publications, *low* willingness to cooperate, and *no* present network recommendation led to low selection recommendations, and here neither applicant gender nor evaluator gender mattered. Accordingly, the first configuration was dependent on evaluator gender and the second configuration led to low selection recommendations independent of gender (applicant and evaluator gender). Those findings indicate that all three success factors (i.e., publications, willingness to cooperate, network recommendation) were taken somewhat into consideration and a network recommendation does not seem to matter for male evaluators in our study.

**Table 3:** Configurations leading to low selection recommendation.

	Configuration	
	1	2
Success factors		
Publications	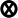	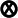
Willingness to cooperate	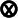	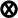
Network recommendation		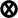
Demographics		
Applicant gender		
Evaluator gender	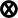	
Consistency	0.906	0.906
Raw coverage	0.270	0.275
Unique coverage	0.129	0.134
Overall consistency: 0.892		
Overall coverage: 0.404		

Circles with “x” indicate the absence of a condition (i.e., publications *solid*, willingness to cooperate *low*, network recommendation *non-present*, applicant gender *male*, and/or evaluator gender *male*). All conditions are core conditions. Blank space indicates a “don’t care” condition.

## Discussion

Shedding light on the subjective interpretation of seemingly objective selection criteria, the aim of this research was to investigate gendered arguments in applicant assessments for STEM professorships. The recruitment contexts promote clearly defined criteria and objective assessment; still, the criteria can be subjectively interpreted and construed differently for men vs. women, leading to gender-biased evaluations (van den Brink and Benschop, 2012; Herschberg et al., 2015). In our research, we illuminate both the influence of subjective heuristics and objectively observable signals in applicant assessments. To test for gender bias despite having objectively the same information cues given for male and female applicants, we explored whether success patterns are gendered.

Our findings indicate several types of gendered arguments which are likely to fuel each other and fuel gendered discussions in appointment committees for STEM professorships. Those include arguments regarding women’s exceptional status, questioning women, and perceived self-questioning of women. Additionally, in a situation in which applicants are comparable regarding publication records, showing willingness to cooperate, and having a network recommendation, we found both gender-independent and gender-dependent success patterns (i.e., “configurations” in fsQCA) for selection recommendation.

### Gendered arguments fueling gendered discussions

Previous research revealed gendered discussions based on a “proven masculine success model” in appointment committees for professorships (van den Brink and Benschop, 2012, 2014, p. 14). Focusing on STEM professorships, which are particularly male stereotyped high-status positions in academia (Carli et al., 2016; Dutz et al., 2022), our findings highlight different forms of gendered arguments despite desired objectivity in applicant assessments; thereby updating and extending our knowledge of the persistence and mechanisms of gendered arguments in appointment committee discussions.

Gendered arguments revealed a paradox of women’s exceptional status in the contexts. Female applicants for STEM professorships seem to be perceived as having a “unique selling point” in recruitment processes (i.e., their gender). However, until they get to the point where gender is perceived to help them get a position, their exceptional status and gender was related to adverse deviance with the male “prototype” of STEM professors and outgroup derogation. The perceptions emphasize, on the one hand, women’s “exceptional” representation and, on the other hand, allegedly required “exceptional” attributes of women (e.g., being assertive “as men,” following a male-typed career track without interruptions, and being visible in male-typed networks despite barriers for women; see, e.g., Heilman, 2012; van den Brink and Benschop, 2014). Thereby, our findings show arguments from evaluator perspective to support a gender authenticity challenge for women in science careers (“unusual” for their career and “unusual” for women; Faulkner, 2007), likely fueled by narratives of their exceptional status (Müller, 2021). Future research needs to consider that exceptional status arguments in discussions of female applicants are complex and may help or hinder gender equality efforts.

Furthermore, questioning women found in our study comprehensively illustrates in the context of STEM professorships how female applicants are not given the benefit of the doubt as (perceived) “risky” options (Fleming Cabrera, 2010; van den Brink and Benschop, 2014). First, in line with status characteristics theory (Foddy and Smithson, 1989; Foschi, 1992), ability questioning indicates that female applicants (as low-status group) need to prove their ability more than male applicants (as high-status group), specifically in STEM where “ability” is male-typed (see also Biernat and Kobrynowicz, 1997). This further relates to a greater potential of ascribing *female* applicants a perceived lack of fit with male-typed job requirements (Heilman, 1983, 2012). Second, general traits as relating to perceived fit, same as abilities (Kristof-Brown, 2000), were subject to questioning regarding perceptions of a “chilly” climate for women in STEM (see e.g., Casad et al., 2021). The focus of arguments was women’s adaption to the climate rather than adapting the climate, presenting a defective “fix the women” solution (Burkinshaw and White, 2017). Last, although female applicants may be perceived in different light associated with the male-typed context if they are perceived as qualified (e.g., possessing male-typed abilities and traits; Eagly, 1987; Dutz et al., 2022), other prejudicial arguments related to social role perceptions, such as women’s (anticipated) care role and alleged lower commitment, can still be influencing perceptions (see also Peterson Gloor et al., 2021). We call for future research to investigate how the questioning arguments influence each other and perceived applicant fit.

The questioning arguments clearly relate to heuristics and stereotyping (Tversky and Kahneman, 1974; Kunda and Thagard, 1996; Heilman, 2012). Of the different types, questioning ability is most likely to be discussed as if it would be “objective,” although likely based on heuristics (Tversky and Kahneman, 1974) and gendered standards (van den Brink and Benschop, 2012; Herschberg et al., 2015). Evaluators may be particularly inclined to question general traits and commitment when the female applicant is perceived to fulfill the general requirements for the job, i.e., is “approved” in terms of evaluated abilities. Both, questioning general traits and commitment, seem to be more recognized as subjective and “informal”; the arguments unavoidably include judgment based on hypothetical considerations, and the higher the ambiguity in applicant assessments, the more likely stereotypes influence perceptions of applicants (Nieva and Gutek, 1980; Heilman, 2012).

Perceived self-questioning of women, further reflected in gendered arguments, connects to *perceived* self-stereotyping of women (Heilman, 2012; Hentschel et al., 2019), e.g., assuming that women generally think they cannot handle the job or are not good enough, therefore engaging in self-limiting behavior. On the one hand, this may indicate a gender-stereotypical image evaluators still have of female applicants (e.g., uncertain and self-critical) vs. ideal scientists (e.g., decisive and high self-regard; Carli et al., 2016). On the other hand, this may indicate a less positive self-impression of women than men in STEM careers. That is, when engaging in self-stereotyping, women may question their “unusual” career track and perceived “gender-atypical” behavior on the job, not perceiving gender authenticity (Faulkner, 2007; Müller, 2021), and thus their fit and commitment to the job (e.g., Heilman, 1983, 2012). Gender authenticity, not feeling the need to explain one’s career choice (Faulkner, 2007), may enhance women’s positive self-impression and reduce self-doubt. Importantly, arguments of perceived self-questioning of women again reflect a “fix the women” rather than “fix the system” approach (Burkinshaw and White, 2017). Interestingly, while self-criticism and low (demonstrated) self-confidence were seen as barriers for women applying to STEM professorships, self-criticism was generally seen as a crucial trait in scientists and being overly self-confident in appointment processes as rather negative, challenging the stereotypical view of ideal scientists (Carli et al., 2016).

### Objectively observable signals and gendered success patterns

We not only investigated gendered arguments, but also which combinations of objectively observable signals or success factors can lead to selection recommendations. Previous literature suggests that, beyond criteria directly signaling academic competence (e.g., publications), there are gender-stereotyped, notably stereotypical female, requirements (e.g., Rehbock et al., 2021). For example, expressing willingness to cooperate and being recommended in one’s scientific network can be important signals of potential academic success incorporating stereotypically female components (see, e.g., Gazdag et al., 2022; Heilman, 2012). The findings of our fsQCA indicated that gender-independent success patterns for selection recommendations, with respect to both applicant and evaluator gender, include not only outstanding publications but also signaling the willingness to cooperate as success factors. Additionally, our results indicated that network recommendations, suggesting interpersonal skills that are stereotypically female (but also stereotypical male self-promotion skills; Rudman, 1998; Heilman, 2012), can make a difference for men and women alike.

Interestingly, applicants without outstanding publications (having only solid publications) were still recommended for selection, when they were either female or had a female evaluator, when they signaled willingness to cooperate and additionally had a network recommendation. Conversely, with a male evaluator, solid publications and low willingness to cooperate led to a *low* selection recommendation for male and female applicants, and here a network recommendation did not matter. Thus, a network recommendation in combination with signaling high willingness to cooperate can become particularly important for female applicants, and female evaluators seem to care about a network recommendation more than male evaluators. In other words: A network recommendation legitimizes female applicants considered to be perceived as “risky” options and female evaluators seem to be particularly aware that such recommendations are needed to be accepted in the community (see van den Brink and Benschop, 2014). However, it was also recognized in our interviews that women are often the “outgroup” to networks in STEM academia and it may be more difficult for women to get a network recommendation (see also van den Brink and Benschop, 2014). Thus, our findings point towards several obstacles but also some success factors particularly for women.

### A mixed methods approach to capture complexity of gendered influences

Our findings on gendered arguments and gendered success patterns, and the subjective interpretation of seemingly objective criteria, emphasize the complexity of gendered influences in appointment committees for STEM professorships. Combining analyses of responses to vignettes and interview questions helped us to understand the more subjective and the more objective parts of applicant assessments, including interviewees’ reasoning behind their survey ratings (see also Einola and Alvesson, 2021), and how applicants may be evaluated based on comparable profiles controlling for influences of gendered arguments. Taking this approach, we are able to contextualize and interpret the survey ratings in light of our interviewees’ verbatim statements highlighting some interesting aspects.

Introducing the assessment scenario and vignettes, we also introduced the proposed selection criteria publications, showing willingness to cooperate, and having a network recommendation. Publication records were often mentioned as important selection criterion beforehand, while publication success was interpreted in light of a continuous track record especially female applicants sometimes may not have due to interruptions for parental leave. Cooperativeness was seen as highly crucial in hypothetical applicants, while not so much discussed before the criteria was introduced in the assessment scenario. Conversely, gendered arguments rather pointed to the fact that such qualities stereotypically more found in women are undervalued in appointment committee decisions. Last, visibility and being well-known in the scientific network were discussed as crucial even before the assessment scenario was introduced, while, in the scenario, the interviewees were tentative to incorporate the criterion of a network recommendation in their evaluations due to its subjectivity. Network recommendations were described as subjective and informal, while still recognizing they are influencing perceptions of applicants when observed for one applicant but not for another. Interestingly, while interviewees further indicated a network recommendation may be difficult to get for women, they valued it especially in women.

The findings indicate that gender stereotypes play a role in many different forms and can be part of heuristics and can also influence judgments based on objectively observable signals. Therefore, to move towards a comprehensive understanding of assessments in appointment committees, influences on different levels, in different domains, and in different stages of the recruitment process need to be considered. Clearly defined and objectively assessable criteria can be highly valuable, particularly in earlier stages of recruitment, while in later discussions additional heuristics and in turn gendered arguments may come to play.

### Practical implications

Although recruitment for STEM professorships generally promotes clearly defined and objectively amenable criteria for assessment and selection, subjective discussions of applicants still make a large part of the process. Adding more structure to those discussions can help to objectify the processes. For instance, appointment committees should discuss and agree on specific criteria *before* sharing information about applicants or starting to discuss their suitability (see, e.g., Heilman, 2012). Not all committee members may have the same information about the position and prior considerations at job advertising (see also van den Brink and Benschop, 2012). STEM professors play a crucial role in creating and promoting a picture of the diverse and actual requirements of the jobs (Rehbock et al., 2021) rather than collapsing into a stereotypical assessment pattern of how they think STEM professors typically are or are expected to be like when evaluating *others’* suitability (see, e.g., Male et al., 2009). With clearly defined criteria and requirements, appointment committees can define the signals to look for in applicants, and the questions asked to obtain information about certain applicant qualities that fulfill the desired profile. For instance, committee members can reflect on whether appearing highly self-confident when presenting one’s achievements is a requirement (e.g., to master demands such as heading executive education), or simply corresponds more with the stereotypical image whereas abilities to be self-critical and self-reflective are the more valuable qualities for scientists.

In addition, training committee members is crucial for them to not only be aware of gendered arguments but also learn ways to recognize and challenge those arguments (see, e.g., Horvath, 2018). For example, the committee chair can assign a trained committee member the role of a devil’s advocate challenging assumptions made about applicants that are not yet verified or lack reliable information cues for verification.

STEM professors are not only part of necessary change as committee members; they can further be role models promoting a diverse image of STEM professors and different possible academic life tracks. This includes normalizing parental leaves, as well as efforts towards a more inclusive work climate in STEM fields such that there is no question that women may feel uncomfortable or have problems integrating. Universities further need to increase their efforts of demonstrating how academia is a good working environment to balance work and family for professors of all genders, actively considering the needs of care takers and providing enough help and structures that align with care taking responsibilities (see, e.g., Greider et al., 2019). In general, there is a need to normalize that women, same as men, are pursuing science (Müller, 2021). The paradox of women’s exceptional status shows that, as long as femininity is perceived as deviance, regulations need to be set and closely monitored to ensure that women are considered in recruitment, while potentially creating a perceived female advantage.

Finally, we can derive recommendations for female applicants for STEM professorships. They cannot directly influence the discussions in appointment committees. However, knowing what aspects are potentially problematized in female more than in male applicants, female applicants can provide information cues as counter signals of questioning their ability, general traits, and commitment. For instance, they can signal they have a realistic job preview and clearly state the abilities and past achievements helping them to meet the requirements; they can describe how they are part of the STEM community and will integrate into the faculty (e.g., describing planned cooperation and committee work); and they can emphasize their commitment to research and teaching. Indeed, prior research showed that a “maybe baby” penalty female applicants are facing is reduced when female applicants emphasize their commitment to work (Peterson Gloor et al., 2021).

Furthermore, our finding that particularly female applicants may profit from a network recommendation highlights that efforts of women to get inroads into networks and work with well-known senior scientists likely pay off. Problematic is that networks in (STEM) academia are often made for and by men and women can only influence to a limited extent whether they are perceived as “being part of it” (van den Brink and Benschop, 2014).

### Limitations and future research

In the current research, we implemented a mixed methods approach to capture both gendered arguments and gendered success patterns. Our approach was particularly qualified to model applicant assessments in a qualitative research approach, while additionally gathering quantitative ratings based on objectively observable applicant attributes. While this approach helped us to understand the full picture of influences of heuristics, stereotyping, and signals, it also has some limitations.

In our exploratory approach we did not test specific hypotheses. However, based on our findings, propositions to be tested in future field survey and experimental research can be derived. For instance, more closely investigating the paradox of women’s exceptional status in STEM careers is of high practical relevance, including the questions of whether a perceived exceptional status is more or less pronounced, or has a different connotation, depending on, e.g., male-typed hobbies of female applicants, the perception of maternity, or prior failures of women in similar positions. Moreover, questioning behavior towards male vs. female applicants needs further investigation in a quantitative study (similar to Kanze et al., 2018).

Furthermore, social desirability may have played a role in our interviews. The interviewee’s and interviewer’s identity were disclosed. Thus, to some extent, our interviewees may have been inclined to answer in a socially acceptable way. Yet, we declared anonymized handling of the gathered data and statements used to illustrate our points will not allow conclusions about individual interview partners. The overall impression further was that most of the interviewed professors talked quite openly about their experiences in appointment committees (while some considered themselves as having a greater gender equality mindset than other appointment committee members typically have). Additionally, for their quantitative ratings, the interviewees filled out a survey that was processed anonymously.

The assessment scenario construed for professors’ ratings was a simplification of reality as a limitation of experimental designs. Using a conjoint experiment, we could look at different applicant attributes simultaneously. However, in practice, applicant attributes and their levels are more complicated than how they were modeled in the conjoint experiment, and applicants are less comparable in reality. Nevertheless, the conjoint experiment can show how objectifying the process, i.e., evaluating applicants on comparable criteria and signals, can foster gender equality. Furthermore, although the interviewees did not make actual decisions on applicants, they considered the applicant attributes to reflect relevant and realistic criteria. Furthermore, due to our think aloud approach, they could add criteria they think are important or share other additional thoughts or comments on applicant evaluations.

Last, the generalizability of our findings to other contexts than STEM professorships may be limited. Although we can draw parallels from (perceptions of) professors to other leaders (Braun et al., 2013; Dutz et al., 2022; Rehbock et al., 2022), some criteria for the assessment and selection of professors do not matter in industry contexts (e.g., publications).

### Conclusion

Recruitment contexts such as recruitment for STEM professorships promote selection criteria clearly defined and objectively assessed; illuminating the subjective interpretation of seemingly objective criteria, and gendered arguments in applicant assessments in theses contexts, our findings corroborate that they are not as objective as they seem. Still, our findings suggest that relying on specific signals that are at least objectively observable can objectify applicant assessments and thus foster gender equality. Importantly however, objectively observable signals need to be carefully defined and still cannot eliminate gender bias completely.

## Data availability statement

The datasets generated for this study are available on request to the corresponding author.

## Ethics statement

Ethical review and approval was not required for the study on human participants in accordance with the local legislation and institutional requirements. The participants provided their written informed consent to participate in this study.

## Author contributions

All authors listed have made a substantial, direct, and intellectual contribution to the work and approved it for publication.

## Funding

This research was funded by the German Federal Ministry of Education and Research–Bundesministerium für Bildung und Forschung (BMBF); Grant Number (FKZ): 01FP1602.

## Conflict of interest

The authors declare that the research was conducted in the absence of any commercial or financial relationships that could be construed as a potential conflict of interest.

## Publisher’s note

All claims expressed in this article are solely those of the authors and do not necessarily represent those of their affiliated organizations, or those of the publisher, the editors and the reviewers. Any product that may be evaluated in this article, or claim that may be made by its manufacturer, is not guaranteed or endorsed by the publisher.

## Footnotes

^1^To avoid a dropout of conditions exactly at 0.5, we added a constant of 0.001 to conditions <1, in our data set (Ragin, 2008b; Fiss, 2011; Pappas and Woodside, 2021).

^2^Therefore, we did not set a frequency threshold to include or exclude cases (see Ragin, 2008a; Fiss, 2011).
